# Assessing Exposure and Health Consequences of Chemicals in Drinking Water: Current State of Knowledge and Research Needs

**DOI:** 10.1289/ehp.1206229

**Published:** 2014-01-03

**Authors:** Cristina M. Villanueva, Manolis Kogevinas, Sylvaine Cordier, Michael R. Templeton, Roel Vermeulen, John R. Nuckols, Mark J. Nieuwenhuijsen, Patrick Levallois

**Affiliations:** 1 Centre for Research in Environmental Epidemiology (CREAL), Barcelona, Spain; 2 IMIM (Hospital del Mar Medical Research Institute), Barcelona, Spain; 3 CIBER Epidemiología y Salud Pública (CIBERESP), Madrid, Spain; 4 National Institute of Public Health, Athens, Greece; 5 Inserm (Institut National de la Santé et de la Recherche Médicale) UMR 1085, IRSET (Institut de Recherche Santé Environnement et Travail), Université de Rennes 1, Rennes, France; 6 Imperial College London, Department of Civil and Environmental Engineering, London, United Kingdom; 7 Environmental Epidemiology Division, Institute for Risk Assessment Sciences (IRAS), Utrecht University, Utrecht, the Netherlands; 8 Department of Environmental and Radiological Health Sciences, Colorado State University, Fort Collins, Colorado, USA; 9 Université Laval, Québec, Québec, Canada; 10 Institut National de Santé Publique du Québec, Québec, Canada; 11 Centre de recherche du Centre hospitalier universitaire (CHU) de Québec, Québec, Canada

## Abstract

Background: Safe drinking water is essential for well-being. Although microbiological contamination remains the largest cause of water-related morbidity and mortality globally, chemicals in water supplies may also cause disease, and evidence of the human health consequences is limited or lacking for many of them.

Objectives: We aimed to summarize the state of knowledge, identify gaps in understanding, and provide recommendations for epidemiological research relating to chemicals occurring in drinking water.

Discussion: Assessing exposure and the health consequences of chemicals in drinking water is challenging. Exposures are typically at low concentrations, measurements in water are frequently insufficient, chemicals are present in mixtures, exposure periods are usually long, multiple exposure routes may be involved, and valid biomarkers reflecting the relevant exposure period are scarce. In addition, the magnitude of the relative risks tends to be small.

Conclusions: Research should include well-designed epidemiological studies covering regions with contrasting contaminant levels and sufficient sample size; comprehensive evaluation of contaminant occurrence in combination with bioassays integrating the effect of complex mixtures; sufficient numbers of measurements in water to evaluate geographical and temporal variability; detailed information on personal habits resulting in exposure (e.g., ingestion, showering, swimming, diet); collection of biological samples to measure relevant biomarkers; and advanced statistical models to estimate exposure and relative risks, considering methods to address measurement error. Last, the incorporation of molecular markers of early biological effects and genetic susceptibility is essential to understand the mechanisms of action. There is a particular knowledge gap and need to evaluate human exposure and the risks of a wide range of emerging contaminants.

Citation: Villanueva CM, Kogevinas M, Cordier S, Templeton MR, Vermeulen R, Nuckols JR, Nieuwenhuijsen MJ, Levallois P. 2014. Assessing exposure and health consequences of chemicals in drinking water: current state of knowledge and research needs. Environ Health Perspect 122:213–221; http://dx.doi.org/10.1289/ehp.1206229

## Introduction

The safety of water supplies is of paramount public health importance. An estimated 13% of the world population lacked access to improved drinking-water sources in 2008 [UNICEF and World Health Organization (WHO) 2011], and almost 10% of the total burden of disease worldwide could be prevented by improving drinking-water supply, sanitation, hygiene, and the management of water resources (Prüss-Üstün et al. 2008). Microbiological contamination is the largest cause of waterborne disease at a global scale. However, chemicals in water supplies can be related to health risks, generally when associated with long-term exposures (Thompson et al. 2007).

There are uncertainties about the safety of current standards for some regulated chemicals, and the potential health impacts of unregulated or emerging chemical contaminants are largely unknown. In May 2012, a workshop was held at the Centre for Research of Environmental Epidemiology (CREAL), Barcelona, Spain, with the aim of advancing the field of epidemiology and chemical contaminants in water and to make recommendations for future research. Our aspiration was that the proposed suggestions be useful and applicable to any type of chemical contaminant occurring in drinking water. Chemicals that we discuss as examples in this review are substances whose main pathway of human exposure is through drinking water. Although the chemical universe is broad and most chemicals do not occur exclusively in drinking water, water is essential for life, and exposures to chemicals in drinking water, even at low concentrations, may have important consequences across the entire population. Here we focus on cancer as an example and summarize the main discussion points and conclusions of the workshop.

## Occurrence

*Regulated chemicals*. Drinking-water quality is regulated in most countries, and monitoring is conducted routinely. A complete list of chemicals that are currently regulated in drinking water, and the regulatory limits promulgated for each chemical by the WHO (2011), the U.S. Environmental Protection Agency (EPA 2009), and the European Union (EU) Council (1998) are provided in Table 1. These regulatory guidelines require periodic review to be updated according to new evidence. For example, the U.S. EPA reduced its maximum contaminant level (MCL) for arsenic from 50 μg/L in 1942 to the current level of 10 μg/L in response to growing scientific evidence of its adverse health effects (Smith et al. 2002). Epidemiological studies have reported associations of trihalomethane (THM) levels in drinking water (a surrogate measure of the disinfection by-product mixture) and bladder cancer (Villanueva et al. 2004) at THM levels lower than the current regulations in the United States and the European Union (80 and 100 μg/L, respectively; Table 1). The current MCL for nitrate was set based on methemoglobinemia among infants, but there is uncertainty concerning the safety of this MCL for chronic effects over longer exposure periods (e.g., on cancer) (Ward et al. 2005). Manganese is a neurotoxin associated with learning disabilities and deficits in intellectual function in children (Zoni and Lucchini 2013). The WHO manganese guideline has been fluctuating from the initial 500 μg/L in 1958 (Ljung and Vahter 2007) to its discontinuation in the current (fourth) edition of the WHO guidelines (WHO 2011). This has generated controversy in the scientific community because the last guideline before discontinuation (400 μg/L) was questionable according to some authors (Ljung and Vahter 2007) and the discontinuation of the manganese guidelines has received criticisms (Frisbie et al. 2012). Although many contaminants are monitored and regulated, the adequacy of the MCL approach is open to debate, in part because these limits are often based on toxicological studies of animals because human studies are not available or are inconclusive.

**Table 1:** Regulatory limits [μg/L (except for asbestos)] for chemicals in drinking water established by the WHO (2011), the U.S. EPA (2009), and the EU Council (1998).

Chemical	WHO	U.S. EPA	EU	Chemical group
Acrylamide	0.5	a	0.1	Organic
Alachlor	20	2	—	Organic
Aldicarb	10	—	—	Organic
Aldrin + dieldrin	0.03	—	—	Organic
Antimony	20	6	5.0	Inorganic
Arsenic	10	10	10	Inorganic
Asbestos (million fibers >10 μm per liter)	—	7	—	Inorganic
Atrazine	100^*b*^	3	—	Organic
Barium	700	2,000	—	Inorganic
Benzene	10	5	1.0	Organic
Benzo[*a*]pyrene	0.7	0.2	0.010	Organic
Berylium	—	4	—	Inorganic
Boron	2,400	—	1,000	Inorganic
Bromate	10	10	10	DBP
Bromodichloromethane	60	—	—	DBP
Bromoform	100	—	—	DBP
Cadmium	3	5	5.0	Inorganic
Carbofuran	7	40	—	Organic
Carbon tetrachloride	4	5	—	Organic
Chloramines (as Cl_2_)	—	4,000	—	Disinfectant
Chlorate	700	—	—	DBP
Chlordane	0.2	2	—	Organic
Chlorine	5,000	4,000	—	Disinfectant
Chlorine dioxide	—	800	—	Disinfectant
Chlorite	700	1,000	—	DBP
Chlorobenzene	—	100	—	Organic
Chloroform	300	—	—	DBP
Chlorotoluron	30	—	—	Organic
Chlorpyrifos	30	—	—	Organic
Chromium (total)	50	100	50	Inorganic
Copper	2,000	13,000	2,000	Inorganic
Cyanazine	0.6	—	—	Organic
Cyanide	—	200	50	Inorganic
2,4-D (dichlorophenoxyacetic acid)	30	70	—	Organic
Dalapon	—	200	—	Organic
2,4-DB (dichlorofenoxybutyric acid)	90	—	—	Organic
DDT (dichlorodiphenyltrichloroethane) and metabolites	1	—	—	Organic
Dibromochloromethane	100	—	—	DBP
1,2-Dibromo-3-chloropropane (DBCP)	1	0.2	—	Organic
1,2-Dibromoethane	0.4	—	—	Organic
Dichloroacetate	50	—	—	DBP
Dichloroacetonitrile	20	—	—	DBP
1,2-Dichlorobenzene (*o*-dichlorobenzene)	1,000	600	—	Organic
1,4-Dichlorobenzene (*p*-dichlorobenzene)	300	75	—	Organic
1,2-Dichloroethane	30	5	3.0	Organic
1,2-Dichloroethene	50	—	—	Organic
1,1-Dichloroethylene	—	7	—	Organic
*cis*-1,2-Dichloroethylene	—	70	—	Organic
*trans*-1,2-Dichloroethylene	—	100	—	Organic
Dichloromethane	20	5	—	Organic
1,2-Dichloropropane	40	5	—	Organic
1,3-Dichloropropene	20	—	—	Organic
Dichlorprop	100	—	—	Organic
Di(2-ethylhexyl) adipate	—	400	—	Organic
Di(2-ethylhexyl) phtalate	8	6	—	Organic
Dimethoate	6	—	—	Organic
Dinoseb	—	7	—	Organic
1,4-Dioxane	50	—	—	Organic
Dioxin (2,3,7,8-TCDD)	—	0.00003	—	Organic
Diquat	—	20	—	Organic
Edetic acid	600	—	—	Organic
Endothall	—	100	—	Organic
Endrin	0.6	2	—	Organic
Epichlorohydrin	0.4	^*a*^	0.10	Organic
Ethylbenzene	300	700	—	Organic
Ethylene dibromide	—	0.05	—	Organic
Fenoprop/Silvex/2,4,5-TP/2-(2,4,5- trichlorophenoxy)propionic acid	9	50	—	Organic
Fluoride	1,500	4,000	1,500	Inorganic
Glyphosate	—	700	—	Organic
Haloacetic acids (HAAs)^*c*^	—	60	—	DBP
Heptachlor	—	0.4	—	Organic
Heptachlor epoxide	—	0.2	—	Organic
Hexachlorobenzene	—	1	—	Organic
Hexachlorobutadiene	0.6	—	—	Organic
Hexachlorocyclopentadiene	—	50	—	Organic
Hydroxyatrazine	200	—	—	Organic
Isoproturon	9	—	—	Organic
Lead	10	15	10	Inorganic
Lindane	2	0.2	—	Organic
Mecoprop	10	—	—	Organic
Mercury	6	2	1.0	Inorganic
4-(2-Methyl-4-chlorophenoxy) acetic acid (MCPA)	2	—	—	Organic
Methoxyclor	20	40	—	Organic
Metolachlor	10	—	—	Organic
Microcystin-LR	1	—	—	Algal toxin
Molinate	6	—	—	Organic
Monochloramine	3,000	—	—	Disinfectant
Monochloroacetate	20	—	—	DBP
Nickel	70	—	20	Inorganic
Nitrate	50,000	45,000	50,000	Inorganic
Nitrilotriacetic acid	200	—	—	Organic
Nitrite	3,000	4,500	500	Inorganic
*N*-Nitrosodimethylamine (NDMA)	0.1	—	—	DBP
Oxamyl (Vydate^®^)	—	200	—	Organic
Pendimethalin	20	—	—	Organic
Pentachlorophenol	9	1	—	Organic
Pesticides	—	—	0.10	Organic
Pesticides (total)	—	—	0.50	Organic
Picloram	—	500	—	Organic
Polychlorinated biphenyls (PCBs)	—	0.5	—	Organic
Polycyclic aromatic hydrocarbons	—	—	0.10	Organic
Selenium	40	50	10	Inorganic
Simazine	2	4	—	Organic
Sodium dichloroisocyanurate/cyanuric acid	50,000/40,000	—	—	Disinfectant
Styrene	20	100	—	Organic
Tertbutylazine	7	—	—	Organic
Tetrachloroethene (tetrachloroethylene)	40	5	—	Organic
Tetrachloroethylene + trichloroethylene	—	—	10	Organic
Thallium	—	2	—	Inorganic
Toluene	700	1,000	—	Organic
Toxaphene	—	3	—	Organic
Trichloroacetate	200	—	—	DBP
1,2,4-Trichlorobenzene	—	70	—	Organic
1,1,1-Trichloroethane	—	200	—	Organic
1,1,2-Trichloroethane	—	5	—	Organic
Trichloroethene/trichloroethylene	20	5	—	Organic
2,4,6-Trichlorophenol	200	—	—	Organic
2,4,5-T (2,4,5-trichlorophenoxyacetic acid)	9	—	—	Organic
Trifluralin	20	—	—	Organic
Trihalomethanes (total)	—	80	100	DBP
Vinyl chloride	0.3	2	0.50	Organic
Xylenes	500	10,000	—	Organic
DBP, disinfection by-product.^***a***^Each water system must certify annually that when it uses acrylamide and/or epichlorohydrin to treat water, the combination of dose and monomer level does not exceed the ­levels specified, as follows: acrylamide = 0.05% dosed at 1 mg/L (or equivalent); epichlorohydrin = 0.01% dosed at 20 mg/L (or equivalent). ^***b***^Includes its chloro-*s*-triazine metabolites. ­^***c***^Includes the sum of monochloroacetic acid, dichloroacetic acid, trichloroacetic acid, monobromoacetic acid, and dibromoacetic acid.				

*Emerging chemical contaminants*. Non-regulated chemicals are of particular concern and constitute a main focus of current research (Richardson and Ternes 2011). Wastewater from human activities may contaminate water supply sources with pharmaceuticals, nanoparticles, consumer products (such as sunscreens), and other contaminants (Table 2), and these chemicals have been identified in drinking water (Ternes 2007). For example, iodinated or nitrogenated disinfection by-products (DBPs) [which are unregulated DBPs that are more toxic than their chlorinated and carbonaceous DBP analogs (Plewa et al. 2008b)] may occur in water supplies at very low concentrations (Plewa et al. 2004, 2008a). Degradation by-products of pharmaceuticals, which may be more toxic than their parent compounds, also have been identified in drinking water (Shen and Andrews 2011). The contribution of drinking water as a source of exposure to perfluorinated chemicals may be as important as dietary intake (Ericson et al. 2008), and evidence suggests that continued human exposure to even relatively low concentrations of perfluorooctanoic acid (PFOA) in drinking water results in elevated body burdens that may increase the risk of health effects (Post et al. 2012). Although concentrations are generally low (usually in the range of nanograms per liter) and some individual chemicals may pose no appreciable risks to human health (Schriks et al. 2010), there are concerns about potential risks of exposures to mixtures (Silva et al. 2002). The removal efficiency by drinking-water treatment processes has been evaluated for some substances (WHO 2012) but is poorly known for many emerging pollutants.

**Table 2:** Emerging chemical contaminants that may occur in water sources or treated drinking water [data from Richardson and Ternes (2011)], with the current state of information regarding their health effects.

Chemical group	Source	Chemicals	Suspected or known health effects
Algal toxins	Produced by algal blooms from an excess of nutrients (in agricultural runoff and wastewater discharges).	Microcystins (e.g., microcystin-LR), nodularins, anatoxins, cylindrospermopsin, and saxitoxins.	Microscystin-LR is hepatotoxic, genotoxic, and carcinogenic (IARC 2010).
Artificial sweeteners	Consumers > urban wastewater > natural waters > drinking-­water source.	Sucralose (Splenda^®^, SucraPlus™), acesulfame, saccharin, cyclamate, etc.	Unknown. Sucralose is a persistent chemical in the environment (half-life up to several years).
Brominated flame retardants	Used during many years in commercial products such as children’s sleepwear, foam cushions in chairs, computers, plastics, and electronics. Diet is a source of exposure because some are persistent and accumulate in fish, eggs, milk, and meat.	Several chemicals classified in different groups such as polybrominated diphenyl ethers (PBDEs), polybrominated biphenyl (PBB), hexabromocyclododecane (HBCD).	Neurotoxicity and thyroid disruption (Dingemans et al. 2011).
Benzotriazoles	Complexing agents widely used as anticorrosives and for silver protection in dishwashing liquids.	The two most common forms are benzotriazole and tolytriazole.	Unknown. Soluble in water, resistant to biodegradation, and only partly removed in wastewater treatment.
DBPs	Generated through chemical reaction between organic matter and a disinfectant (e.g., chlorine, chloramine, chlorine dioxide) in the treatment of drinking water and swimming pools.	More than 700 compounds identified to date, which together are estimated to account for ~ 50% of the total organic halogen content.	Genotoxic, carcinogenic, reprotoxic.
Ionic liquids	Organic salts with low melting point (< 100ºC) promoted as “green chemistry” replacements to traditional solvents in industry. They exhibit some unique properties, including tunable viscosity, miscibility, and electrolytic conductivity, which make them useful for many applications, including organic synthesis and catalysis, production of fuel cells, batteries, coatings, oils, and nanoparticles, as well as other chemical engineering and biotechnology applications.	The chemical structures typically involve a cationic or anionic polar head group with accompanying alkyl side chains. Cationic head groups include imidazolium, pyridinium, pyrrolidinium, morpholinium, piperidium, quinolinium, quaternary ammonium, and quaternary phosphonium moieties; anionic head groups include tetrafluoroborate (BF_4_^–^), hexafluorophosphate (PF_6_^–^), bis(trifluoromethylsulfonyl)-imide [(CF_3_SO_2_)_2_N^–^], dicyanamide [(CN)_2_N^–^], chloride, and bromide.	Different toxicity in animals (Pham et al. 2010). No human studies.
Illicit drugs	Found in surface waters, but generally removed by treatment in water utilities (Huerta-Fontela et al. 2008).	Several chemicals, including amphetamine-like compounds, benzodiazepines, cannabinoids, cocainics, lysergic acid diethylamine (LSD), opioids, and metabolites (Valcárcel et al. 2012).	The effect of the mixture is unknown.
Musks	Highly lipophilic chemicals widely used as fragrance additives in many consumer products including perfumes, lotions, sunscreens, deodorants, and laundry detergents.	Several chemicals. May have nitroaromatic structures [as in the case of musk xylene (1-*tert*-butyl-3,5-dimethyl-2,4,6-trinitrobenzene) or musk ketone (4-*tert*-butyl-2,6-dimethyl-3,5-dinitroacetophenone)] or polycyclic structures [as in the case of 7-acetyl-1,1,3,4,4,6-hexamethyl-1,2,3,4-tetrahydronaphthalene (AHTN; trade name, tonalide), 1,3,4,6,7,8-hexahydro-4,6,6,7,8,8-hexamethylcyclopenta-(*g*)-2-benzopyran (HHCB; trade name, galaxolide), 4-acetyl-6-*tert*-butyl-1,1-dimethylindan (ADBI; trade name, celestolide), dihydropentamethylindanone (DPMI; trade name, cashmeran), or 5-acetyl-1,1,2,3,3,6-examethylindan (AHMI, trade name phantolide)].	Endocrine disruption, according to animal evidence (Schreurs et al. 2004).
Naphtenic acids	Result from petroleum extraction. Occur naturally in crude oil deposits across the world (up to 4% by weight) and in coal.	Complex mixture of alkyl-substituted acyclic and cyclo-aliphatic carboxylic acids that dissolve in water at neutral or alkaline pH and have surfactant-like properties.	Liver toxicity in mammals (Rogers et al. 2002). No human studies.
Nanomaterials	Heterogeneous group of chemicals sized 1–100 nm, highly stable, strong, conductors, and with low permeability.	Several chemical groups and structures including fullerenes, nanotubes, quantum dots, metal oxanes, titanium dioxide, nanoparticles, nanosilver, and zerovalent iron nanoparticles.	Unknown.
Perfluorinated compounds (PFCs)	Used to make stain repellents (such as Teflon), and in the manufacture of paints, adhesives, waxes, polishes, metals, electronics, fire-fighting foams, and caulks as well as grease-proof coatings for packaging. Diet is the main route of exposure, followed by drinking water, house dust, and air.	Different types. The most common are perfluorooctanoic acid (PFOA) and perfluorooctanesulfonic acid (PFOS).	Liver, pancreatic, and testicular tumor in animals. Inmunotoxicity (DeWitt et al. 2012), thyroid function disruption (Boas et al. 2012; Melzer et al. 2010).
Pesticide transformation by-products	Result from the hydrolysis, oxidation, biodegration, or photolysis of pesticides. Can be present at higher levels than the parent compound and can be as toxic or more toxic. Diet is a source of exposure.	Several chemicals, such as alachlor ethanesulfonic acid (ESA), alachlor oxanilic acid (OA), acetochlor ESA, acetochlor OA, metolachlor ESA, metolachlor OA, 3-hydroxycarbofuran, and terbufos sulfone.	Unknown.
Pharmaceuticals	Human consumption > excretion > urban wastewater > natural waters > drinking-­water source.	Several chemicals, including antidepressants, antiviral drugs, glucocorticoids, antimycotics, antibiotics, beta-blockers.	The effect of the mixture is unknown.
Siloxanes	Used in cosmetics, deodorants, soaps, hair conditioners, hair dyes, car waxes, baby pacifiers, cookware, cleaners, furniture polishes, and water-repellent windshield coatings.	Cyclic siloxanes [octamethylcyclotetrasiloxane (D4), decamethylcyclopentasiloxane (D5), dodecamethylcyclohexasiloxane (D6), and tetradecamethylcycloheptasiloxane (D7)] and linear siloxanes.	Unknown.
Sunscreens/ultraviolet filters	Personal care products > urban wastewater > natural waters > drinking-­water source. Identified in drinking water (in Barcelona, Spain) with average concentrations up to 295 ng/L (Díaz-Cruz et al. 2012).	Several chemicals. The ones identified in drinking water are benzophenone-3 (BP3), octocrylene (OC), 2-ethylhexyl 4-methoxycinnamate (EHMC), 3-(4-methylbenzylidene) camphor (4-MBC), and 2-ethylhexyl 4-(dimethylamino) benzoate (OD-PABA).	Unknown.
Single chemicals
Dioxane	High-production chemical used as a solvent stabilizer in the manufacture and processing of paper, cotton, textile products, automotive coolants, cosmetics, and shampoos and as a stabilizer of 1,1,1-trichloroethane (a degreasing agent).	1,4-Dioxane. Regulated by U.S. EPA (50 mg/L).	Unknown.
Perchlorate	Highly stable and soluble chemical used in solid propellants in rockets, missiles, and fireworks as well as in highway flares. Can be found as a contaminant in sodium hypochlorite. Perchlorate can accumulate in plants and has been found in biological samples.	Perchlorate	Unknown. Perchlorate can cross the placenta.

## Global Indicators of Toxicity

Water supplies often include mixtures of chemical contaminants that vary in time and space. In addition, the epidemiological and toxicological evaluation of mixtures involves significant challenges, in many cases beyond the limits of current research methods. *In vitro* bioassays (or biosensors) developed through toxicological research are promising tools for measuring the global toxicity of chemical mixtures in water samples and may be coupled with more in-depth analysis of specific contaminants when a positive response is detected. For example, Jeong et al. (2012) evaluated *in vitro* mammalian cell toxicity for a range of DBPs in an attempt to identify specific DBPs responsible for genomic DNA damage. End points that can be measured by *in vitro* bioassays include mutagenicity (Ames test) (Richardson et al. 2010), genotoxicity (micronuclei, Comet assay) (Plewa et al. 2010), endocrine disruption (DR-CALUX bioassay) (Brand et al. 2013; Sato et al. 2010), and cytotoxicity (Plewa et al. 2010). Although the use of these markers is not without limitations (such as the need for complex and nonstandardized sample pretreatment methods in order to obtain concentrates before laboratory analysis and the uncertain validity for some of the assays, limited throughput development, elevated cost, low sensitivity, and results reflecting only short-term exposure evaluations). Further development of these techniques and their incorporation into epidemiological research may improve our understanding of the effects of mixtures. These efforts will require improved, interdisciplinary communication and collaboration including analytical chemists, toxicologists, and epidemiologists.

## Human Exposure

Accurate exposure assessment in human observational studies is essential to obtain valid results and constitutes a main methodological challenge, as summarized in Table 3. Difficulties in identifying and measuring contaminants in water supplies at very low concentrations and substances occurring in mixtures hamper the evaluation of human exposure, requiring new methods in health risk analysis (Schwarzenbach et al. 2006).

**Table 3:** Challenges of exposure assessment for chemical contaminants in drinking water.

Challenge	Comments
Low exposure levels	Accuracy of analytical measurements in water is particularly important at the low range of exposure. In addition, detailed personal information of water use behavior is convenient.
Chemicals occurring in mixtures	Examples include pharmaceutical residues and disinfection by-products. Depending on the individual constituents of the mixture, chemical-by-chemical exposure assessment may not be feasible or could result in simplistic exposure estimates.
Time–space variability	Repeated measurements and distribution of sampling points covering different water zones is necessary to evaluate geographical and temporal variation during the relevant exposure period.
Long-term exposure windows	Longer exposure periods are likely to result in greater exposure misclassification. In the case of chronic diseases such as cancer, data collection must include accurate location of study participants (residence and workplace) and water use over the duration of an exposure period relevant to disease etiology. Combined with environmental levels, quantitative estimation of exposure can be conducted. An added challenge is the lack of historical monitoring data.
Lack of monitoring data	This is particularly problematic to evaluate some exposures (such as emerging contaminants) and some outcomes (such as cancer because historical records are frequently unavailable). More research is needed to develop validated simulation models that can be used to estimate levels and exposure over the relevant time period.
Lack of validated biomarkers of exposure	Currently available validated biomarkers typically reflect recent exposures and thus may not be useful for outcomes with latency periods longer than the half-life of the biomarker compound. Exceptions may occur if the time between consecutive exposure events is shorter than the elimination half-life or exposure can be regarded as constant within the relevant time window (such as for trichloroacetic acid).
Multiple exposure routes (ingestion, inhalation, dermal absorption)	Exposure to a number of water contaminants can occur through multiple routes. For example, some DBPs can be incorporated through inhalation, dermal absorption and ingestion. For other waterborne contaminants, such as nitrate (at levels in water < 50 mg/L) and per- and polyfluorinated compounds, diet is the main source of exposure (Ericson Jogsten 2011; IARC 2010). For such contaminants, exposure by all plausible routes should be assessed in order to produce the most accurate estimate of disease risk.

DBPs are an example of chemicals occurring in complex mixtures, and this has been addressed in part by using a few compounds as surrogates for the DBP mixture as a whole. For example, observational studies of human DBP exposures and health effects have focused on a small subset of the several hundred DBPs that may occur in public water supplies (Richardson et al. 2007), particularly the THMs and haloacetic acids (HAAs) (Hinckley et al. 2005; Hoffman et al. 2008; Righi et al. 2012). However, although these compounds are often used as a surrogate for other DBPs, the assumption that they correlate with other DBPs is not universally supported, and correlations can vary in time and space (Villanueva et al. 2012).

Methods of exposure assessment are influenced by the specific outcome under study. For instance, for end points with a long latency, such as cancer, long time periods of more than several decades need to be evaluated, whereas for reproductive outcomes, it is very important to accurately capture the temporal variation in exposure over a shorter period covering the relevant time windows before and during gestation.

Chemicals or metabolites have been measured in biological samples in epidemiological studies to estimate exposures [e.g., urinary or toenail arsenic measurements in cancer studies (Karagas et al. 2004)]. Urinary trichloroacetic acid is a promising biomarker of DBPs that requires methodological development before a generalized use in epidemiological studies (Savitz 2012). In addition, among the available biomarkers specific for drinking-water contaminants, many have short half-lives (e.g., urinary trichloroacetic acid) and are thus of limited value to associate with health outcomes that require long-term exposures (Savitz 2012). Consequently, exposure assessment in most instances relies on assessing personal behavior (ascertained through questionnaires) and measuring environmental levels of the chemicals (Hoffman et al. 2008; Levallois et al. 2012).

Inhalation and dermal contact may be relevant exposure routes for volatile or skin-permeable chemicals. In such cases, activities involving different water uses at home (e.g., showering, bathing), in recreation (e.g., swimming in pools), and through occupations involving water contact should be considered.

Alternative methods of exposure assessment may involve statistical modeling; for example, modeling based on known geographic distributions of contaminants (Toledano et al. 2005), hydrological modeling of underground plumes of contaminants (Gallagher et al. 2010), and/or the use of surrogate parameters such as land use (Aschebrook-Kilfoy et al. 2012). Several methods can be used in combination, tailored to the availability of data; for example, in a recent study on the long-term exposure to arsenic and cancer, Nuckols et al. (2011) combined arsenic data from their own measurements in water samples collected at homes of the participants, data from public water utilities, and historical data for aquifers.

Exposure estimates with minimal measurement error are necessary to produce valid effect estimates. Misclassification of exposure is of particular concern at the low exposure range because it tends, under most scenarios, to attenuate associations toward the null (Cantor and Lubin 2007; Waller et al. 2001) or to reduce the precision of associations (Wright and Bateson 2004). Strategies to minimize measurement error are necessary from study design to data analysis, and include, for example, the collection of repeated measures of individual water use over the relevant exposure period (Forssen et al. 2009) and assessing reliability of interviews to exclude unreliable questionnaires (Villanueva et al. 2009).

## Health Effects

The following is an overview of epidemiological findings from individual-based studies of chemical contaminants in water and cancer. Table 4 displays a summary of the evidence of carcinogenicity as evaluated and concluded by the WHO International Agency for Research on Cancer (IARC).

**Table 4:** Evidence of carcinogenicity as concluded by the IARC for some chemicals whose main pathway of human exposure is through drinking water [modified from the General Remarks to IARC Monograph, Volume 101 (IARC 2012b)].

Agent	Human evidence	Animal evidence	Overall evaluation^*a*^ (group)	*IARC Monograph*
Elements
Arsenic	Sufficient	Sufficient	1	Vol. 100 C (IARC 2012a)
Fluoride	Inadequate	Inadequate	3	Suppl. 7 (IARC 1987)
Nitrate	Inadequate	Inadequate/sufficient^*b*^	2A^*c*^	Vol. 94 (IARC 2010)
Microcystin-LR	Inadequate	Inadequate	2B	Vol. 94 (IARC 2010)
DBPs: Trihalomethanes
Chloroform	Inadequate	Sufficient	2B	Vol. 73 (IARC 1999)
Bromodichloromethane	Inadequate	Sufficient	2B	Vol. 52 (IARC 1991)
Dibromochloromethane	Inadequate	Limited	3	Vol. 52 (IARC 1991)
Bromoform	Inadequate	Limited	3	Vol. 52 (IARC 1991)
DBPs: Haloacetic acids
Dichloroacetic acid	Inadequate	Sufficient	2B	Vol. 106 (IARC 2013)
Trichloroacetic acid	Inadequate	Sufficient	2B	Vol. 106 (IARC 2013)
Bromochloroacetic acid	Inadequate	Sufficient	2B	Vol. 101 (IARC 2012b)
Dibromoacetic acid	Inadequate	Sufficient	2B	Vol. 101 (IARC 2012b)
DBPs: Halogenated acetonitriles
Bromochloroacetonitrile	No data	Inadequate	3	Vol. 52 (IARC 1991)
Chloroacetonitrile	No data	Inadequate	3	Vol. 52 (IARC 1991)
Dibromoacetonitrile	No data	Inadequate	3	Vol. 52 (IARC 1991)
Dichloroacetonitrile	No data	Inadequate	3	Vol. 52 (IARC 1991)
Trichloroacetonitrile	No data	Inadequate	3	Vol. 52 (IARC 1991)
Dibromoacetonitrile	No data	Sufficient	2B	Vol. 101 (IARC 2012b)
Chloral hydrate	Inadequate	Sufficient	2A	Vol. 106 (IARC 2013)
MX (3-chloro-4-(dichloromethyl)-5-hydroxy-2(5H)-furanone)	Inadequate	Limited	2B^*d*^	Vol. 84 (IARC 2004)
Bromate (evaluated as potassium bromate)	Inadequate	Sufficient	2B	Vol. 73 (IARC 1999)
Chlorite (evaluated as sodium chlorite)	No data	Inadequate	3	Vol. 52 (IARC 1991)
Chlorinated drinking water	Inadequate	Inadequate	3	Vol. 52 (IARC 1991)
Chemicals used in the disinfection of drinking water
Hypochlorite salts	No data	Inadequate	3	Vol. 52 (IARC 1991)
Chloramine	Inadequate	Inadequate	3	Vol. 84 (IARC 2004)
^***a***^Group 1 (the agent is carcinogenic to humans), 2A (the agent is probably carcinogenic to humans), 2B (the agent is possibly carcinogenic to humans), 3 (the agent is not classifiable as to its carcinogenicity to humans). ^***b***^There is sufficient evidence in experimental animals for the carcinogenicity of nitrite in combination with amines or amides. ^***c***^Ingested nitrate or nitrite under conditions that result in endogenous nitrosation is probably carcinogenic to humans. ^***d***^Other relevant data were used to upgrade the evaluation.				

There is sufficient evidence in humans that arsenic in drinking water causes cancers of the urinary bladder, lung, and skin (IARC 2004). Studies conducted in areas with lower levels of arsenic in drinking water (i.e., at or below the MCL) have reported inconsistent results, and cancer risks associated with exposure to low arsenic levels over decades remain uncertain.

Bladder cancer has been consistently associated with DBP exposure (Cantor 2010), and pooled analyses combining data from studies conducted in different countries have reported associations between bladder cancer and THM at levels below current MCLs (Costet et al. 2011; Villanueva et al. 2004). Some (Cragle et al. 1985; King et al. 2000; Wilkins and Comstock 1981), but not all (Doyle et al. 1997; Hildesheim et al. 1998; Koivusalo et al. 1997), studies of DBP exposure and colon cancer have reported positive associations. Similarly, positive associations for DBP exposure have been found for rectal cancer (Bove et al. 2007; Doyle et al. 1997; Hildesheim et al. 1998) not replicated in other studies (King et al. 2000; Koivusalo et al. 1997; Wilkins and Comstock 1981).

The epidemiological investigation for nitrate and cancer has been challenging. Drinking water may be a primary source of nitrate exposure when drinking-water concentrations are > 50 mg/L (IARC 2010). Below this threshold, diet is the main exposure route, involving complex mechanisms of action through endogenous formation of *N*-nitroso compounds (IARC 2010). Long-term exposure to nitrate in drinking water has been evaluated in relation to multiple cancer sites including the esophagus, stomach, bladder, and colon (IARC 2010). Although there is inadequate human evidence for carcinogenicity, there is sufficient evidence from experimental animals for the carcinogenicity of nitrite in combination with amines or amides, and ingested nitrate under conditions that result in endogenous nitrosation has been classified as probably carcinogenic to humans (IARC 2010).

Other contaminants have been less extensively investigated in relation to cancer risk. Fluoride is added to drinking water at low concentrations in some countries to prevent dental caries, and naturally occurs in water at higher levels in certain parts of the world such as the Rift Valley in Africa (Malde et al. 2011). The IARC (1987) evaluated fluoride carcinogenicity and concluded that human and animal evidence was inadequate (Table 3). Some epidemiological studies on osteosarcoma have been published after this evaluation (Bassin et al. 2006; Kim et al. 2011), but consistent associations have not been observed.

The liver is a target organ for microcystin-LR (IARC 2010), which are toxins produced by cyanobacteria as a result of algae blooms and the eutrophication of surface waters. Individual-based studies evaluated by IARC (2010) have assessed exposure by comparing water consumed from ponds or ditches versus other sources and no measurements of toxins or bacteria were considered. In consequence, IARC concluded that evidence in humans for the carcinogenicity of microcystin-LR is inadequate (IARC 2010). Other carcinogens such as heavy metals, pesticides, and solvents may occur in drinking water as a consequence of human activities and natural hydrogeochemical processes. However, evidence on the cancer risk on human populations is limited.

## Mechanisms and Biomarkers

The elucidation of mechanisms of action to provide biological plausibility and support causality suggested by epidemiological associations is a priority in current research. Biomarkers of early effect can be used in epidemiological studies to provide evidence about subclinical or intermediate effects of exposures (e.g., cytogenetic changes), and effects of very low exposure levels, and they can be used in experimental studies to evaluate the effect of an intervention. For an intermediate biomarker to be informative, it should be associated with both the disease and exposure of interest and reflect an intermediate step in the pathway between exposure and disease. For example, a suggested mechanism of action for arsenic is through epigenetic dysregulation, although there are limited human studies available (Ren et al. 2011). In addition, the evaluation of genetic variants may be used to identify susceptible populations underlying the biological mechanisms of action. For example, the evaluation of genetic variants of DBP-metabolizing enzymes in an epidemiological study on bladder cancer and THM exposure has shown that polymorphisms in key metabolizing enzymes modified DBP-associated bladder cancer risk (Cantor et al. 2010). In addition, the consistency of these findings with experimental observations of GSTT1 (glutathione *S*-transferase theta 1), GSTZ1 (glutathione *S*-transferase zeta 1), and CYP2E1 (cytochrome P450, family 2, subfamily E, polypeptide 1) enzymatic activity strengthens the hypothesis that DBPs cause bladder cancer and suggests possible mechanisms, as well as the classes of compounds likely to be implicated (Cantor et al. 2010).

There are few validated biomarkers specific for chemical contaminants in drinking water. However, the availability of prospective studies with biobanked samples and biotechnological development allowing large numbers of compounds to be measured in small amounts of biological samples (e.g., urine, plasma, serum) is encouraging. These technologies include genomics, epigenomics, transcriptomics, adductomics, proteomics, and metabolomics (Rappaport and Smith 2010; Wild 2005). Application of these techniques will facilitate a comprehensive approach to identify perturbations in biological systems and associated mechanisms of action (Moore et al. 2013). These technologies have not been widely applied in water research but have shown promising results in other areas of environmental research.

## Future Challenges

A significant and growing body of evidence suggests that climate change will have a detrimental effect on the quality of water available for human consumption in the future. For example, increasing temperatures may enhance conditions for the proliferation of cyanobacteria and algae (Joehnk et al. 2008; Newcombe et al. 2012; Paerl and Huisman 2008). Cyanobacteria are of particular concern for human populations because they can produce cyanotoxins such as microcystin that have carcinogenic effects (IARC 2010). The frequency of extreme weather events is expected to increase as a consequence of climate change, and the concentrations of chemical contaminants may be affected by extreme precipitation events. For example, tests conducted in models of different types of soils showed that certain mobile pharmaceuticals occur at higher concentrations in soil and groundwater during and directly after intense precipitation events (Oppel et al. 2004). Simulation studies have shown that pesticide concentrations fluctuate with changes in precipitation intensity and seasonality (Bloomfield et al. 2006; Probst et al. 2005). Evidence concerning the effect of drought is mixed. For example, concentrations of heavy metals (e.g., chrome, mercury, lead, cadmium) introduced primarily from anthropogenic activities in the Rhine River basin are higher during drought years (Zwolsman and van Bokhoven 2007). In contrast, no significant changes during drought conditions, but significant variability between seasons, has been described in the Dommel River, a tributary of the Meuse river in the Netherlands where increased groundwater flow in winter led to increased metal concentrations (Wilbers et al. 2009). In summary, it is expected that climate change could adversely affect drinking-water quality, but there is limited knowledge concerning the magnitude and distribution of the impact at different scales (global, regional, local).

## Final Remarks and Recommendations

*General aspects*. Although microbiological contamination is the largest contribution to waterborne disease and mortality at a global scale, chemical contaminants in water supplies also can cause disease, sometimes after long periods of exposure. The concentrations in drinking water, the prevalence of human exposure in the population, and the level of toxicity can be used to prioritize chemicals for further research. These characteristics may vary geographically and, therefore, further research should be designed to local-, region-, or country-specific circumstances as appropriate. Finally, exposures and risks affecting vulnerable populations (e.g., children and pregnant women) require special attention and are of particular interest.

Arsenic is a unique example of a substance in drinking water with conclusive evidence from human epidemiological studies. There is no doubt that arsenic is a human carcinogen at high concentrations (IARC 2004); however, there is inadequate information to determine the carcinogenic potential of other chemicals that occur in drinking water (Table 4). Arsenic has several unique characteristics—including the fact that drinking water represents the predominant source of exposure in humans; the levels in water, and thus the magnitude of the exposure, is very high in certain areas (e.g., Bangladesh); the availability of measurements in drinking water has allowed the development of epidemiological studies; the wide variability in exposures facilitates the detection of risks; the occurrence as an isolated substance rather than in mixtures allows the direct measurement of the putative agent; the magnitude of the risks are high compared with other chemicals; and the existence of biomarkers—all of which have helped to improve exposure assessment and elucidation of mechanisms of action of arsenic.

*Recommendations on occurrence and exposure assessment*. Improved exposure assessment to water contaminants is essential to derive valid exposure–response curves and useful knowledge for risk assessment and regulation, and here we provide some suggestions.

The research need concerning regulated chemicals is to clarify the effects at or below their MCLs, which are suspected for some contaminants. Access to water utility monitoring data, which is necessary to conduct such studies, should be encouraged and facilitated. Access to large databases would facilitate improved exposure assessment in epidemiological studies, if the data are reliable and sufficient to evaluate temporal and geographical variations applicable to study areas.
The measurement of emerging contaminants needs advanced and specialized analytical methods, and close collaboration between epidemiologists and analytical chemists is required to provide contaminant occurrence data suitable in format and quantity for epidemiological research. Better communication between epidemiologists and environmental analytical chemists would facilitate human health studies in this area. A mechanism to converge interests might be to collect water samples for analytical chemistry method development alongside ongoing epidemiological studies, or training analytical chemists in exposure assessment procedures.
The evaluation of mixtures requires some attention in future studies because this remains a challenge beyond current methods. New developments may contribute to understand the health effects of chemical contaminants in drinking water.
Some *in vitro* assays as indicators of water toxicity are promising tools deserving incorporation in future studies to complement exposure assessment and health risk analyses. These bioassays may be especially effective to evaluate the global effect of chemical mixtures and identify “hot spots” of toxicity. Such findings can be useful in generating hypotheses for more in-depth and resource-intensive analysis of specific contaminants and health outcomes. Incorporating these methods in epidemiological research should be encouraged, and further validation should be conducted when necessary.
Epidemiological research generally requires large numbers of measurements and data. This may constitute a challenge in the collaboration with analytical chemists and toxicologists if experimental methods are manual or laborious but should be overcome in the future with, for example, the development of high-throughput techniques able to analyze large amounts of water samples.
Ongoing cohort studies should be encouraged to incorporate a water dimension because retrospective assessment is challenging, particularly for outcomes with a long latency such as cancer. This would require water sample collection, measurements, and personal questionnaires in ongoing cohort studies, and new or reinforced collaborations between research groups. New cohorts (or data collections in existing cohorts) should be also encouraged to implement environmental sampling and storage of such samples (envirobanking) for use in future nested case–control studies.
Methods developed for environmental and geospatial sciences, including geographical information systems and fate/transport modeling of chemicals, have been demonstrated to be useful in exposure assessment for risk analysis for waterborne chemical contaminants. Consequently, greater emphasis on incorporating these methodologies into environmental epidemiological studies should be made.
Climate change is likely to affect water quality with uncertain implications for human health. Research to evaluate these impacts and the potential human health consequences at different regional scales and in different climates is necessary.

*Recommendations on epidemiological methods*. Epidemiological studies based on rigorous study design are essential to properly evaluate the human health risks associated with chemical contaminants in drinking water. Here we summarize some suggestions in this direction.

There is a need to investigate the potential health outcomes of emerging (i.e., non-regulated) contaminants because current knowledge on health effects is mainly limited to regulated chemicals. However, there are still uncertainties and further research is needed to evaluate potential effects below MCLs for certain regulated chemicals.
Studies capturing widely contrasting exposure levels are particularly useful to estimate risks. Therefore, environmental epidemiologists should influence the decision as to the location of study sites on this basis.
Large studies with sufficient statistical power are necessary when the expected health risks are small in magnitude. It is advisable to know contaminant levels and exposure prevalence before undertaking an epidemiological study to allow the estimation of sample size to reach sufficient statistical power.
The incorporation of biomarkers of exposure, effect, and genetic susceptibility in epidemiological studies is encouraged to identify molecular mechanisms of action and to contribute to the assessment of causality. Studies evaluating biomarkers could be companion studies within ongoing larger or small- to medium-sized experimental studies. In particular, -omic technologies can add to the current understanding of biological mechanisms and generate new hypotheses, requiring advanced and complex statistical tools to deal with the large amounts of data generated. However, biomarkers must be validated and biomarker studies generally require large numbers of observations and replication in multiple populations. Additional drawbacks of biomarker studies are the relatively high cost, the limitation of biomarkers with regard to capturing past exposures, their invasiveness, and the possibility for reverse causation (i.e., in cross-sectional or case–control studies).

*General conclusions*. Assessing the health impacts of chemical contaminants in drinking water is a challenge that requires improved methodologies and enhanced interdisciplinarity in future epidemiological studies. Useful and valuable knowledge will increase if future studies successfully integrate existing and new developments from analytical chemistry, toxicology, exposure science, molecular epidemiology, statistics, environmental epidemiology, environmental sciences, engineering, and geospatial sciences. Improved cooperation and collaboration with stakeholders such as the water industry, regulatory, and public health agencies and affected communities would serve to produce higher-quality risk analyses, as well as to improve the likelihood of implementing effective and early intervention measures. Institutional support promoting access to reliable routine monitoring data at all levels and collaboration with stakeholders (e.g., water utilities, regulators, and consumer groups) would be beneficial. Finally, research efforts in this area are frequently hampered by the lack of specific funding for this research field, and the availability of stable and substantial financial support is needed, either from governmental or nongovernmental sources.
